# Incidence and risk factors of urinary tract infection in hospitalized patients with spinal cord injury in a hospital of China

**DOI:** 10.1038/s41598-024-54234-2

**Published:** 2024-02-13

**Authors:** Jiawei Liu, Xiaoxia Hao, Xingru Shang, Ruimin Chi, Tao Xu

**Affiliations:** Department of Rehabilitation, Tongji Hospital of Tongji Medical College of Huazhong University of Science and Technology, Jiefang Avenue, No. 1095, Wuhan, 430030 Hubei China

**Keywords:** Incidence, Prevention, Risk factors, Spinal cord injury, Urinary tract infection, Risk factors, Urethra, Trauma

## Abstract

Urinary tract infection (UTI) caused by spinal cord injury (SCI) can have significant morbidity. There is currently a lack of relevant data in China. This study explores incidence and risk factors of UTI in hospitalized patients with SCI in China, and will help healthcare professionals to make informed clinical decisions to reduce the incidence of UTI. This retrospective study analyzed the medical records of patients with SCI who were hospitalized at three campuses of a hospital in central China between August 2014 and August 2023. The files of patients with SCI were reviewed for demographics and clinical characteristics. Logistic regression analysis was performed to identify risk factors associated with UTI. A total of 538 patients were included in this study. The incidence of UTI was 49.8%. Sex, hypoproteinemia, urinary incontinence, bladder irrigation, timing of rehabilitation, duration of indwelling urinary catheter were risk factors of UTI. The implementation of specific preventive measures is anticipated to result in a decrease in the occurrence of UTI among individuals with SCI, consequently enhancing their overall quality of life and prognosis.

## Introduction

The global incidence of spinal cord injury (SCI) ranges from 3.6 to 195.4 per million people and is increasing annually^1^. Urinary tract infection (UTI) is the most common infection in patients with SCI and a common cause of hospitalization^2^. UTI affects the patient's rehabilitation because it usually increases spasticity and may even require bed rest as well as prolonged hospitalization^3^. In a population-based survey conducted in Switzerland, 59% of patients with SCI developed UTI complications with 41% describing the complications as moderate or severe^4^. UTI reduces patients' quality of life, and can lead to hydronephrosis, acute kidney injury, urologic tumor development, sepsis, or renal failure, all of which can severely reduce patients' lives^5,6^.

Due to loss of sensation, the signs and symptoms of UTI in patients with SCI, such as urinary frequency, urgency, and difficulty in urination are not specific. They sometimes may only have objective symptoms, such as chills and fever, leading to an often inability to make an early diagnosis^7^. Antibiotics can be efficacious in prevention and treatment; however repeated administration of antibiotics increases the risk of multi-drug resistance^8^.

With the development of rehabilitation medicine technology in recent years, catheterization techniques and materials for patients with SCI have been significantly improved, and urinary problems have been systematically managed. However, the incidence of UTI in patients with SCI is still high. A recent literature review, reported that UTI is common in patients with SCI, with a reported prevalence of 10–68% globally^9^. Different countries have reported different incidences of UTI suggesting that the incidence varies widely depending on the medical setting and patient characteristics. As a developing country with a large population, UTI in patients with SCI in China is of concern. However, China lacks a national SCI registry system. Therefore, there were no definitive results on the incidence of UTI in Chinese SCI patients. There were no previous reports on the analysis of risk factors associated with UTI in Chinese patients with SCI either. Exploring the associated risk factors will help healthcare professionals to make informed clinical decisions to effectively prevent and manage UTI in patients with SCI. Therefore, this retrospective study was conducted to investigate the incidence of UTI in hospitalized Chinese patients with SCI and to analyze the factors affecting the occurrence. The primary objective was to gain insight into the present condition and requirements of medical and nursing care for UTI in this specific patient population, with the ultimate aim of devising a more targeted approach for disease intervention.

## Material and methods

### Study population

**Figure 1:**
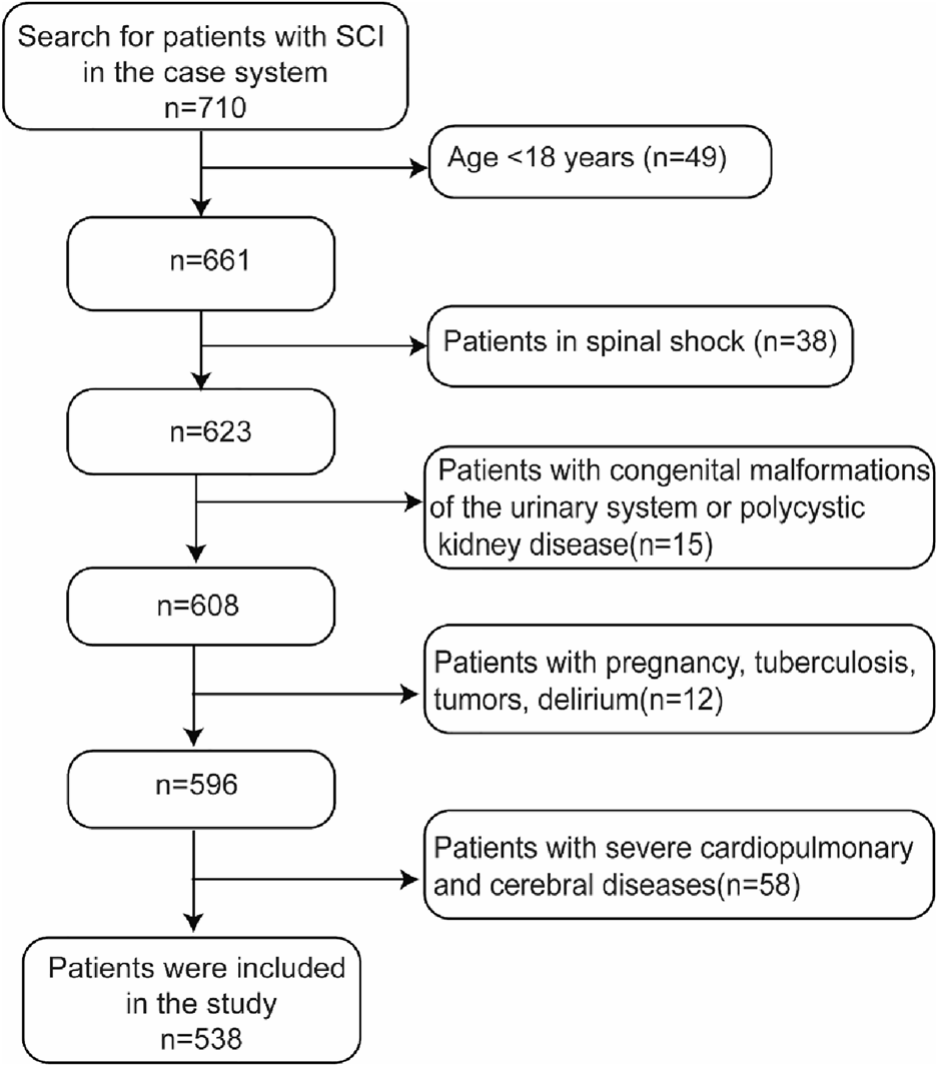
Flow chart for sampling.

We analyzed the medical records of patients with SCI hospitalized in the Department of Rehabilitation Medicine, Tongji Hospital, Tongji Medical College, Huazhong University of Science and Technology, China, between August 2014 and August 2023. This is a Grade 3A general hospital located in the central region of China, encompassing three medical centers dispersed across various regions of Wuhan. Grade 3A hospitals are the highest level of hospitals in mainland China, and are hospitals above the regional level that provide high-level medical and health services to multiple regions and perform higher education and scientific research tasks. Tongji Hospital has more than 7000 beds and treats patients from all over the country. The number of patients with SCI treated annually is over 200, and the admission rate is over 60%. The rehabilitation department of this hospital holds the sixth position within China's rehabilitation sector and functions as the Hubei Provincial Rehabilitation Quality Control Center. The data from this study may represent a better level of SCI management in China and may be generalizable to SCI management in other parts of China. The study included patients diagnosed with SCI, according to the International Classification of Diseases Version 10 (ICD-10: T09.300). Figure 1 shows the flow chart for sampling.

The Ethics Committee of Tongji Hospital, Tongji Medical College, Huazhong University of Science and Technology reviewed and approved this study (Ethics No. TJ-IRB202402063). Our study has been performed in accordance with the Declaration of Helsinki. We confirm that all methods were performed in accordance with the relevant guidelines and regulations. The need for individual informed consent for this retrospective study was waived.

### Patient and public involvement statement

Patients or the public were not involved in the design, or conduct, or reporting, or dissemination plans of our research.

### Exclusion criteria

Age < 18 years.

Patients in spinal shock: During spinal shock, the patient's nervous system is in an unstable state, and many ratings are inaccurate^10^. Additionally, there are differences in treating the disease during spinal shock compared to managing the non-shock period^11^. These factors can impact the accuracy of the study.

Patients with congenital malformations of the urinary system or polycystic kidney disease: UTI are more likely to occur in this condition due to structural abnormalities in the urinary tract or impaired kidney function^12^. These factors can impact the accuracy of the study.

Patients with pregnancy, tuberculosis, tumors, delirium, severe cardiopulmonary and cerebral diseases: Many changes occur in the patient's body during pregnancy, including hormone levels and metabolic rate. Tumors may cause an abnormal response of the immune system. Severe cardiopulmonary and cerebral diseases encompass a range of conditions such as myocardial infarction, heart failure, respiratory failure, stroke, craniocerebral injury, and other conditions that can lead to impaired heart, lung, and brain function that may affect the patient's physiological state and metabolic function^13^. Therefore, to ensure the accuracy and reliability of the results, these factors that may affect the results were excluded from this study.

### Variables and definitions

Variables were selected based on published studies, accessibility, and professional knowledge. Variables included were: age, sex, etiology, injury completeness, neurological level, hypoproteinemia, urinary incontinence, bladder irrigation, timing of rehabilitation, duration of indwelling urinary catheter, and Barthel Index scores^14–17^.

UTI were defined in accordance with the International Spinal Cord Society Urinary Tract Infection Basic Data Set^18^. UTI is defined as the new onset of symptoms accompanied by laboratory findings (bacteriuria, leukocyturia, and positive urine culture). Unlike UTI in people without other medical conditions, signs and symptoms of suspected UTI in people with SCI include fever, the first occurrence of urinary incontinence or increased frequency of urinary incontinence (including leakage of urine between the indwelling catheter and the urethra), increased spasmodic state, Suprapubic pain, malaise, hematuria without other causes, lethargy or sensory disturbances, cloudy urine with increased odor, discomfort or pain in the renal/bladder area, difficulty in urinating, or abnormal autonomic reflexes^18,19^.

The extent of injury was based on the American Spinal Injury Association impairment scale (AIS). Serum albumin < 30 g/L was considered hypoproteinemia. Symptoms or urodynamic studies were used to diagnose urinary incontinence. Bladder irrigation was determined by whether bladder irrigation had been performed before UTI. The timing of rehabilitation was calculated from the onset of injury to the time the patient started the rehabilitation program. (Including exercise training, physical therapy, bladder management, etc.). Duration of indwelling urinary catheter stands for the total number of days of voiding with an indwelling catheter before the first UTI in a patient with SCI.

### Statistical analysis

Microsoft Excel (version 16.0) and IBM SPSS Statistics (version 23.0) were used to perform statistical analysis. Data entry was done independently by two different researchers. One researcher entered the collected data into Excel spreadsheets, while the other researcher checked the entered data to eliminate bias and data entry errors, ensuring the comprehensiveness and accuracy of the data. Categorical nominal variables were presented as percentages and analyzed using Pearson’s chi-square test or Fisher’s exact test. Since all continuous variables in this study were non-normal distributed, they were presented as medians and interquartile ranges (IQR) And the nonparametric test (Mann–Whitney U test) was used for analysis. .Logistic regression analysis was used to determine the factors affecting UTI, and odds ratios (OR) and 95% confidence intervals (CI) were calculated. *P*-value < 0.05 was considered statistically significant; *P*-value < 0.01 was considered highly statistically significant.

### Ethics approval and consent to participate

This study was reviewed and approved by the Ethics Committee of Tongji Hospital, Tongji Medical College, Huazhong University of Science and Technology (Ethics No.TJ-IRB202402063). Our study has been performed in accordance with the Declaration of Helsinki. We confirm that all methods were performed in accordance with the relevant guidelines and regulations.

## Results

This study comprised a sample size of 538 participants, with UTI occurring in 268 subjects. Thus, the incidence of UTI was 49.8%. Table 1 shows the characteristics of the patients. Among the patients, 78.6% (n = 423) were men and the median age was 45 years (IQR 21). The primary cause of SCI was traffic accidents (n = 137). The distribution of completeness, based on the AIS, revealed that grade A, B, C, and D injuries constituted 43.8% (n = 235), 22.3% (n = 120), 22.5% (n = 121), and 11.4% (n = 61) of the total cases, respectively. There were 107(19.9%) patients had urinary incontinence and 172(32.0%) patients underwent bladder irrigation. The median number of days between injury and the start of rehabilitation of the participants was 21 days (IQR 14). The median duration of the indwelling urinary catheters was 34 days (IQR 21). The median Barthel Index scores for the patients was 15 (IQR 15).

**Table 1:** Basic characteristics of the UTI and non-UTI groups.

Variable	Categories	Total (n = 538)	UTI (n = 268)	Non-UTI (n = 270)
		N (%) or Median (IQR)	N (%) or Median (IQR)	N (%) or Median (IQR)
Age		45 (21)	45 (21)	44.5 (21)
Sex	Female	115 (21.4)	46 (17.2)	69 (25.6)
	Male	423 (78.6)	222 (82.8)	201 (74.4)
Etiologies	Traffic accident	137 (25.5)	63 (23.5)	74 (27.4)
	High fall	201 (37.4)	99 (36.9)	102 (37.8)
	Low fall	74 (13.8)	42 (15.7)	32 (11.9)
	Hit by object	65 (12.1)	43 (16.0)	22 (8.1)
	Non-traumatic	61 (11.3)	21 (7.8)	40 (14.8)
Completeness of injury	A	235 (43.8)	128 (47.8)	107 (39.8)
	B	120 (22.3)	70 (26.1)	50 (18.6)
	C	121 (22.5)	57 (21.3)	64 (23.8)
	D	61 (11.4)	13 (4.9)	48 (17.8)
Neurological level	Cervical	225 (41.8)	122 (45.5)	103 (38.1)
	Thoracic	242 (45.0)	117 (43.7)	125 (46.3)
	Lumbosacral	71 (13.2)	29 (10.8)	42 (15.6)
Hypoproteinemia	No	438 (81.4)	191 (71.3)	247 (91.5)
	Yes	100 (18.6)	77 (28.7)	23 (8.5)
Urinary incontinence	No	431 (80.1)	185 (69.0)	246 (91.1)
	Yes	107 (19.9)	83 (31.0)	24 (8.9)
Bladder irrigation	No	366 (68.0)	158 (59.0)	208 (77.0)
	Yes	172 (32.0)	110 (41.0)	62 (23.0)
Duration of indwelling urinary cathetertion		34 (21)	41 (26)	29 (18)
Timing of rehabilitation		21 (14)	23.5 (21)	20 (11)
Barthel Index scores		15 (15)	15 (20)	10 (20)

*UTI* urinary tract infection, *IQR* interquartile rang.

Table 2 presents the symptoms and laboratory findings of SCI patients with UTI This study identified the prevailing symptoms observed in patients with SCI during UTI episodes, namely fever, hematuria, and turbid urine. Additionally, a subset of patients reported suprapubic pain, new onset urinary incontinence, and scrotal pain. The most common laboratory findings were leukocyturia, positive urine culture and bacteriuria. Table 3 shows the distribution of microorganisms isolated from urine cultures of SCI patients with UTI. It can be noticed that the most common microorganisms were E. coli.

**Table 2:** Symptoms and laboratory findings in SCI patients with UTI.

	N	Percentage (%)
Symptoms		
Fever	245	91.4
Hematuria	48	17.9
Turbid urine	32	11.9
Suprapubic pain	25	9.3
New onset incontinence	21	7.8
Scrotal pain	12	4.5
Laboratory findings		
Leukocyturia	251	93.7
Positive urine culture	204	76.1
Bacteriuria	105	39.2
Hematuria	97	36.2
Urine nitrite positive	84	31.3
Albuminuria	45	16.8

**Table 3:** Distribution of microorganisms isolated from urine cultures of SCI patients with UTI.

Microorganism	N	Percentage (%)
*E. coli*	82	38.7
*Klebsiella* spp.	36	17.0
*Enterococcus* spp.	31	14.6
*Pseudomonas* spp.	19	9.0
*Acinetobacter* spp.	15	7.1
*Candida* spp.	11	5.2
*Staphylococci* spp.	8	3.8
*Proteus* spp.	6	2.8
*Corynebacterium* spp.	3	1.4
*Xanthomonas* spp.	1	0.5

**Figure 2:**
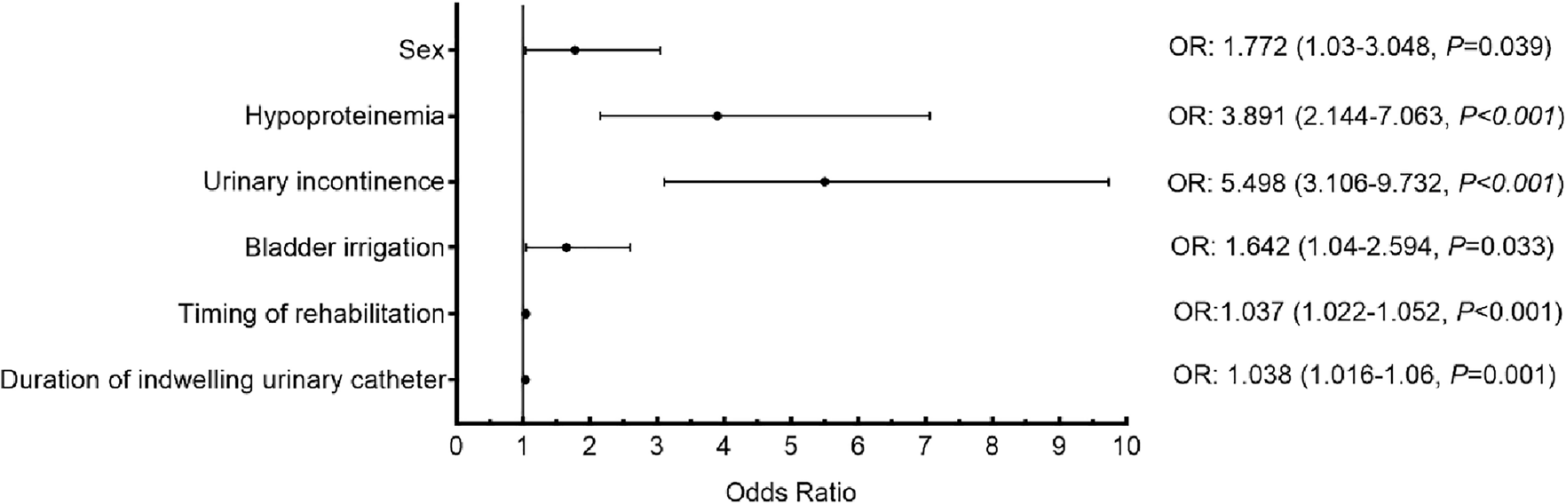
Forest plot for odds ratio with 95% confidence intervals multivariable analysis of risk factors of UTI in patients with SCI.

Table 4 and Fig. 2 shows the results of multivariable regression analyses. We found that sex (OR = 1.772; 95% CI 1.03–3.048), hypoproteinemia (OR = 3.891; 95% CI 2.144–7.063), urinary incontinence (OR = 5.498; 95% CI 3.106–9.732), bladder irrigation (OR = 1.642; 95% CI 1.04–2.594), timing of rehabilitation (OR = 1.037; 95% CI 1.022–1.052), duration of indwelling urinary catheter (OR = 1.038; 95% CI 1.016–1.06) were risk factors of UTI. We further performed stratified analysis by sex (Table 5). Male have a higher rate of UTI than female, and they also have indwelling urinary catheters for a longer period of time compared to female.

**Table 4:** Multivariable logistic regression analysis for factors associated with UTI.

Variable	Categories	B	SE	OR	95%CI	*p*
Age		− 0.015	0.008	0.985	0.97–1.001	0.071
Sex	Female	Reference category				
	Male	0.572	0.277	1.772	1.03–3.048	0.039
Etiology	Traffic accident	Reference category				
	High fall	0.021	0.272	1.022	0.6–1.74	0.937
	Low fall	0.687	0.352	1.987	0.996–3.965	0.051
	Hit by object	0.636	0.388	1.888	0.882–4.042	0.102
	Non-traumatic	− 0.405	0.407	0.667	0.3–1.48	0.319
Completeness of injury	A	Reference category				
	B	0.387	0.28	1.472	0.851–2.546	0.167
	C	− 0.094	0.314	0.91	0.492–1.683	0.764
	D	− 0.719	0.457	0.487	0.199–1.192	0.115
Neurological level	Cervical	Reference category				
	Thoracic	− 0.439	0.273	0.644	0.377–1.101	0.108
	Lumbosacral	− 0.194	0.376	0.824	0.394–1.722	0.606
Hypoproteinemia	No	Reference category				
	Yes	1.359	0.304	3.891	2.144–7.063	< 0.001
Urinary incontinence	No	Reference category				
	Yes	1.704	0.291	5.498	3.106–9.732	< 0.001
Bladder irrigation	No	Reference category				
	Yes	0.496	0.233	1.642	1.04–2.594	0.033
Duration of indwelling urinary cathetertion		0.036	0.008	1.037	1.022–1.052	< 0.001
Timing of rehabilitation		0.037	0.011	1.038	1.016–1.06	0.001
Barthel index scores		− 0.004	0.008	0.996	0.98–1.012	0.598

*CI* confidence interval, *OR* odds ratio, *SE* standard error.

**Table 5:** Stratifying analysis of sex and risk factors of UTI.

Variable	Categories	Female	Male	*p*
		N (%) or Median (IQR)	N (%) or Median (IQR)	
UTI	No	69 (60.0)	201 (47.5)	0.018
	Yes	46 (40.0)	222 (52.2)	
Hypoproteinemia	No	94 (81.7)	344 (81.3)	0.919
	Yes	21 (18.2)	79 (18.7)	
Urinary incontinence	No	88 (76.5)	343 (81.1)	0.277
	Yes	27 (23.5)	80 (18.9)	
Bladder irrigation	No	77 (67.0)	289 (68.3)	0.781
	Yes	38 (33.0)	134 (31.7)	
Duration of indwelling urinary cathetertion		26 (24)	35 (19)	< 0.001
Timing of rehabilitation		22 (12)	21 (14)	0.184

*UTI* urinary tract infection, *IQR* interquartile rang.

**Figure 3:**
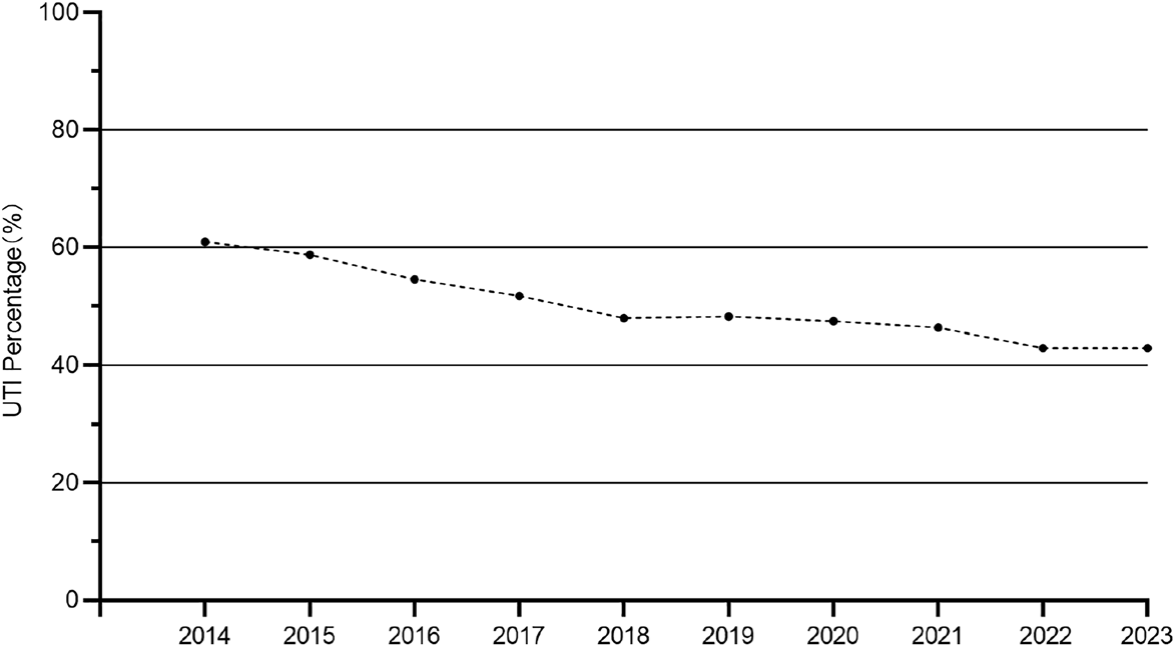
The annual incidence of UTI in patients with SCI from 2014 to 2023.

Figure 3 and Table 6 characterize the annual incidence of UTI and associated factors for each year from 2014 to 2023. As can be seen in Fig. 3, there is a decreasing trend in incidence rates. Table 6 shows that the proportion of hypoproteinemia and urinary incontinence are also decreasing. Notably, since 2020, no bladder irrigation has been performed on patients. At the same time, the duration of the indwelling catheter was progressively shorter, and rehabilitation interventions for patients were earlier (Fig. 3).

**Table 6:** Characteristics of patients with SCI from 2014 to 2023.

		2014	2015	2016	2017	2018	2019	2020	2021	2022	2023
UTI	No	9 (39.1)	26 (41.3)	30 (45.5)	29 (48.3)	25 (52.1)	29 (51.8)	30 (52.6)	36 (53.7)	32 (57.1)	24 (57.1)
	Yes	14 (60.9)	37 (58.7)	36 (54.5)	31 (51.7)	23 (47.9)	27 (48.2)	27 (47.4)	31 (46.3)	24 (42.9)	18 (42.9)
Sex	Female	3 (13.0)	15 (23.8)	15 (22.7)	18 (30.0)	7 (14.6)	8 (14.3)	11 (19.3)	17 (25.4)	13 (23.2)	8 (19.0)
	Male	20 (87.0)	48 (76.2)	51 (77.3)	42 (70.0)	41 (85.4)	48 (85.7)	46 (80.7)	50 (74.6)	43 (76.8)	34 (81.0)
Hypoproteinemia	No	15 (65.2)	46 (73.0)	50 (75.8)	47 (78.3)	38 (79.2)	47 (83.9)	48 (84.2)	58 (86.6)	51 (91.1)	38 (90.5)
	Yes	8 (34.8)	17 (27.0)	16 (24.2)	13 (21.7)	10 (20.8)	9 (16.1)	9 (15.8)	9 (13.4)	5 (8.9)	4 (9.5)
Urinary incontinence	No	17 (73.9)	49 (77.8)	53 (80.3)	47 (78.3)	38 (79.2)	45 (80.4)	46 (80.7)	55 (82.1)	46 (82.1)	35 (83.3)
	Yes	6 (26.1)	14 (22.2)	13 (19.7)	13 (21.7)	10 (20.8)	11 (19.6)	11 (19.3)	12 (17.9)	10 (17.9)	7 (16.7)
Bladder irrigation	No	5 (21.7)	23 (36.5)	25 (37.9)	8 (13.3)	31 (64.6)	52 (92.9)	57 (100.0)	67 (100.0)	56 (100.0)	42 (100.0)
	Yes	18 (78.3)	40 (63.5)	41 (62.1)	52 (86.7)	17 (35.4)	4 (7.1)	0 (0.0)	0 (0.0)	0 (0.0)	00.0)
Duration of indwelling urinary cathetertion		39 (16)	35 (14)	35.5 (22)	34 (34)	34.5 (19)	34.5 (20)	35 (24)	32 (22)	27 (26)	26 (17)
Timing of rehabilitation		24 (14)	23 (8)	22 (17)	22.5 (16)	20 (15)	19 (16)	21 (13)	22 (12)	15 (20)	18 (16)

*UTI* urinary tract infection.

## Discussion

Since China is an important developing country, the study aimed to investigate the prevalence and risk factors of UTI in Chinese hospitalized patients with SCI. Given the significance of UTI prevention in the medical management of patients with SCI, this study holds substantial value. The findings presented herein can aid healthcare teams in acquiring a more comprehensive comprehension of the specific needs of this vulnerable population in China and in implementing appropriate preventive strategies to alleviate the incidence of UTI and associated complications.

### Incidence of UTI in patients with SCI

A recent literature review has indicated that the global prevalence of UTI in patients with SCI ranges from 10 to 68%^9^. This prevalence demonstrates significant variation across different countries, implying that the incidence of UTI is heavily influenced by the healthcare environment and patient-specific factors. In our own study, we observed a prevalence of 49.8%. The findings from these reports and the outcomes of this study indicate that UTI remain prevalent among individuals with SCI and exhibit a higher incidence rate in China. This suggests the need for further development of targeted measures to effectively mitigate the occurrence of UTI.

The prevalent symptoms of UTI in the general populace encompass urinary frequency, urgency, dysuria, and low back pain. Nevertheless, these symptoms may not be readily discernible to individuals with SCI, who may encounter difficulties in accurately articulating their discomfort. The findings of this study indicate that fever is the most common and predominant symptom (> 90%) observed among SCI patients during UTI episodes. Consequently, healthcare professionals should be alert to the possibility of a UTI when a patient presents with fever^7^.

The strains and drug resistance observed in the urine of patients with SCI may exhibit variations compared to those found in able-bodied patients^20^. Urine culture tests serve as a valuable tool for healthcare professionals in confirming the diagnosis of UTI and determining appropriate and efficacious treatment strategies. However, the present study reveals that urine culture was conducted in only 76.1% of patients with UTI, indicating a lack of appropriate utilization in the management of patients with SCI in China^21^. Because urine culture testing allows for early diagnosis, guides treatment, and monitors efficacy, it should be performed when a patient develops new symptoms and suspects a UTI. Based on the test results, we can guide the use of antibiotics. To develop a targeted UTI prevention and treatment program, we also collected microbial distributions from urine cultures. As can be seen in Table 3, the most common microorganism was E. coli (38.7%), followed by Klebsiella spp. (17.0%) and Enterococcus spp. (14.6%). This result is similar to the results of other studies^22,23^.

### Relationship between sex and UTI

This study showed that sex is a factor that affects UTI. This is inconsistent with previous studies, which reported that sex was not associated with UTI^14^. There is a view that women are underrepresented among patients with SCI, therefore, it is difficult to compare the effect of sex on complications, including UTI^24^. Therefore, the findings regarding sex factors on SCI are limited to making incomplete comparisons. Additionally, we conducted a stratified analysis based on sex, revealing that males exhibited a prolonged duration of indwelling catheters compared to females across all identified UTI risk factors. A Korean study also suggests that this may be a result of sex being associated with a higher percentage of male patients having indwelling catheters than female patients, instead of a correlation between UTI and sex^15^. There are significant sex-stratified differences in bladder management after SCI, which should be noted by healthcare professionals.

### Relationship between hypoproteinemia and UTI

Our study showed that hypoproteinemia is a risk factor for UTI in patients with SCI. Human serum albumin is a unique multifunctional protein provides essential fatty acids for neural membrane repair, induces neuroprotection and suppress inflammatory responses after SCI^25–27^. A decreased albumin indicates a reduced ability to resist infection^27,28^. Hypoproteinemia affects the repair of the spinal cord and the immunity of the body, leading to an increased incidence of UTI^27^. In this study, the prevalence of hypoproteinemia in Chinese patients with SCI was as high as 18.6% (n = 100). These data suggest healthcare professionals should be concerned about hypoproteinemia while preventing and treating UTI in patients with SCI. Regular blood tests play a crucial role in the prevention of hypoproteinemia among patients with SCI, while ensuring adequate protein supplementation serves as the primary preventive measure against hypoproteinemia^28^.

### Relationship between urinary incontinence and UTI

Like the findings of previous studies, this study showed that urinary incontinence is a risk factor for UTI in patients with SCI^29^. In this study, the prevalence of urinary incontinence in Chinese patients with SCI was as high as 19.9% (n = 107). Urinary incontinence in patients with SCI is often caused by overactivity of the forced urinary muscles^30^. Overactivity of the detrusor muscle may lead to mucosal ischemia, and inadequate tissue perfusion affecting the natural protection of the mucosa by blood and reducing the release of inflammatory cells, thus promoting the proliferation of microorganisms^31^. Nevertheless, it is imperative to acknowledge that this study is deficient in terms of comprehensive data regarding the absence of urodynamic evaluations among Chinese patients with SCI, as such examinations are not routinely conducted. Consequently, the incapacity to ascertain the specific classification of neurogenic bladder in these patients represents a limitation of this study. This underscores the significance of conducting urodynamic assessments to determine the type of bladder dysfunction in patients afflicted with neurogenic bladder within our clinical practice. Furthermore, early identification of the etiology of urinary incontinence in individuals with SCI and the provision of targeted interventions to effectively manage incontinence symptoms assumes paramount importance.

### Relationship between bladder irrigation and UTI

This study showed that bladder irrigation is a significant risk factor for UTI in patients with SCI. Excessive bladder irrigation can produce mechanical damage to the bladder wall, damaging the bladder mucosa and increasing the risk of infection^32^. It is believed that bladder irrigation instead produces more pathogenic strains of bacteria and increases the risk of infection^33^. However, several studies have shown that effective pharmacologic bladder irrigation for specific UTI can improve treatment outcomes. Linezolid use as a bladder irrigation may be a feasible route of administration in anuric, critically ill patients with VREfm and few antimicrobial options^34^ Bladder irrigation with amphotericin B can improve the curative effect of fungal infection in the urinary tract^35^. Therefore, patients with SCI should avoid unnecessary bladder irrigation and pay attention to aseptic operations when performing bladder irrigation to reduce the risk of UTI.

### Relationship between timing of rehabilitation and UTI

This study showed that the timing of rehabilitation is a risk factor for UTI in patients with SCI. Early comprehensive rehabilitation treatment builds a reflex bladder, establishes regular filling and emptying, induces the formation of an autonomous voiding rhythm, and restores the patient's autonomous voiding function^36^. Early psychological rehabilitation interventions can help patients to understand neurogenic bladder correctly, build confidence in overcoming the disease, reduce disease stigma, improve patient cooperation, and avoid UTI^37^. This prompts healthcare professionals to initiate early rehabilitation in order to mitigate the probability of UTI.

### Relationship between duration of indwelling urinary catheter and UTI

The findings of this study indicate a positive correlation between the duration of indwelling catheterization and the increased probability of developing UTI. Previous study have shown that patients have a 3–7% risk of developing a catheter-associated UTI, per extra day the indwelling urinary catheter remains in place^38^. EAU Guidelines on neurogenic lower urinary tract dysfunction state that indwelling transurethral catheterization should be avoided because it is a risk factor for UTI and significant long-term complications^39^. If the patient's vital signs are unstable or there are other compound injuries, the main focus is to save life and save other organ functions, and a urinary catheter can be temporarily placed. There is no consensus on when to remove the catheter^40^. Nevertheless, based on the extensive body of evidence elucidated above, it is strongly recommended that healthcare professionals expedite the removal of indwelling catheters for the optimal benefit of the patient.

### The incidence and management of UTI in various time periods

Between 2014 and 2023, there is a decreasing trend in the incidence of UTI, which can be attributed to alterations in the healthcare approach towards managing the condition in individuals with SCI. First of all, it is clear that from 2020 no bladder irrigation was performed, which contributed to reduce the incidence of UTI. At the same time, a reduction in the percentage of patients with urinary incontinence and hypoproteinemia can be observed. In addition, rehabilitation interventions became earlier and urinary catheter retention became shorter. These factors collectively contribute to a decrease in the occurrence of UTI. All of these data suggest that the healthcare professionals in the hospitals of this study are more scientific and correct in managing the disease in patients with SCI. Therefore, it is very important to study the risk factors of UTI and manage the disease correctly. In China, the prevalence of UTI in SCI patients is as high as 49.8%, in which case healthcare professionals should pay more attention to the needs of the disease and learn the proper disease management in order to prevent the occurrence of UTI.

## Limitation

This study is a retrospective analysis, which inevitably has many limitations. Firstly, since retrospective studies are based on existing data, there may be bias in the data collected or certain variables may be affected by other factors that may bias the results of the analysis, and therefore there may be problems such as information bias. Secondly, as retrospective studies usually involve the analysis of existing data and information, the choice of researcher may affect the conclusions. Although two researchers were used to collect data in this study, the issue of selection bias in retrospective studies is still unavoidable. Third, urodynamics is an important test for patients with SCI for determining bladder type, and as a reference for bladder management and rehabilitation. Unfortunately, because urodynamics is not widely used in Chinese SCI patients, only some patients in this study obtained urodynamic results, which is a non-negligible limitation. It is recommended in clinical work to improve the urodynamics of patients, which is critical for bladder management and diagnosis. Fourth, although we collected data from SCI patients in the three hospital districts of our hospital, only a small fraction of all SCI patients was identified, and it was not possible to determine the exact prevalence and disease management status of SCI patients in China. Fifth, this study was a single-hospital study, which may bring selection bias and decrease the representativeness of the sample. Sixth, although the hospitals in this study represent the better management of SCI in China and are of some reference value, they may not be generalizable to all hospitals.

## Conclusions

Our findings suggest that the incidence of UTI in Chinese hospitalized patients with SCI was as high as 49.8%, which needs to be paid great attention by clinicians. We found that sex, hypoproteinemia, urinary incontinence, bladder irrigation, timing of rehabilitation, duration of indwelling urinary catheter were risk factors of UTI. Overall, it is of great significance to develop targeted preventive and management measures for these risk factors, which are expected to reduce the incidence of UTI in hospitalized SCI patients in China, and to improve patients' quality of life and rehabilitation outcomes.

## Data Availability

The data that support the findings of this study are available from the corresponding author upon reasonable request.
